# Increased malignancy risk in patients with lymphangioleiomyomatosis: findings from a Chinese cohort

**DOI:** 10.1186/s13023-025-03834-w

**Published:** 2025-05-31

**Authors:** Xiaoxin Zhang, Chongsheng Cheng, Hanghang Wang, Junya Liu, Jiapeng Zhao, Xinhe Zhang, Xinyao Li, Kai-Feng Xu

**Affiliations:** 1 Department of Pulmonary and Critical Care Medicine, State Key Laboratory of Complex, Severe and Rare Diseases, Peking Union Medical College Hospital, Chinese Academy of Medical Sciences & Peking Union Medical College, #1 Shuaifuyuan Hutong, Beijing, China; 2 Chinese Academy of Medical Sciences & Peking Union Medical College, Beijing, 100730 China

**Keywords:** Lymphangioleiomyomatosis, Malignant tumor, Incidence

## Abstract

**Background:**

Lymphangioleiomyomatosis (LAM) is a rare, low-grade neoplasm. Abnormal activation of the mammalian target of rapamycin (mTOR) pathway plays a critical role in LAM pathogenesis by promoting cell proliferation, which may increase susceptibility to malignancies in these patients. However, owing to the rarity of LAM, comprehensive data on the risk of malignancy in this population are limited.

**Methods:**

We retrospectively analyzed 849 LAM patients who participated in the LAM-China Registry Study at Peking Union Medical College Hospital. We collected medical records of patients with malignant tumors and estimated the incidence of malignancy in the LAM-China cohort.

**Results:**

A total of 849 patients were included in our research, of whom 760 were sporadic LAM and 89 had tuberous sclerosis complex-associated LAM. Thirty-one patients (3.65%) had a history of malignancy. More than 80% of LAM and malignant tumors developed successively within 5 years, with a median time interval of 1.5 years. Eighteen patients who were diagnosed with malignancy after diagnosis of LAM were included in the incidence calculation. The three most prevalent cancers were thyroid cancer (*n* = 5), breast cancer (*n* = 4) and ovarian cancer (*n* = 3). Except for lung cancer, the SIRs for all other cancers mentioned in the present study were significantly increased: thyroid cancer (SIR = 10.87, 95% CI 3.53–25.37), breast cancer (SIR = 5.95, 95% CI 1.62–15.24), and ovarian cancer (SIR = 24.54, 95% CI 5.07–71.86). After standardization by age, the SIR for malignancy in our cohort was 3.20 (95% CI 1.89–5.05, *p* = 0.00003). However, this elevated risk of malignancy appeared to be confined to younger age groups. Among individuals aged over 50 years, there was no statistically significant difference in the incidence of malignancy between LAM patients and the reference population.

**Conclusion:**

The risk of malignant tumors is significantly increased in LAM patients than that in the reference population. Thyroid cancer, breast cancer, and ovarian cancer were the three most prevalent malignancies in our cohort. Each type of cancer that appeared in the cohort presented a relatively high incidence, except for lung cancer.

**Supplementary Information:**

The online version contains supplementary material available at 10.1186/s13023-025-03834-w.

## Introduction

Lymphangioleiomyomatosis (LAM) is a rare, low-grade neoplasm that almost exclusively occurs in women, especially those of childbearing age. It is characterized by diffuse cystic lesions in the lungs and symptoms such as pneumothorax and chylothorax or abdominal neoplasms in the kidney, liver or retroperitoneal space [1, 2]. The lesions are caused by LAM cells, which carry mutations in the *TSC1/TSC2* gene, leading to loss of function. LAM is currently believed to be a metastatic tumor, with LAM cells originating from an unknown part of the body and metastasizing to the lungs and abdomen [3].

LAM can also be associated with tuberous sclerosis complex (TSC), a genetic disease. TSC patients carry germline *TSC1/TSC2* gene mutations; thus, this type of LAM is referred to as TSC-LAM. Other LAM patients, called sporadic LAM (S-LAM) patients, only have mutant *TSC1/TSC2* genes in their LAM cells [4, 5]. The loss of function of the *TSC1/TSC2* gene leads to abnormal activation of the mammalian target of rapamycin (mTOR) pathway downstream, which promotes cell growth and proliferation while inhibiting catabolism. It is also a downstream pathway of several cancer-related signaling pathways, such as the MAPK pathway and PI3K/Akt pathway [6].

Research on malignancies in LAM has focused primarily on specific cancer types. Data from two multi-national studies demonstrated that LAM patients have a higher incidence of breast cancer, especially women under the age of fifty [7, 8]. Recent evidence from Japan shows that LAM is also a potential risk factor of lung cancer [9]. However, the studies focused mainly on a specific type of cancer, and comprehensive data on the risk of malignancy in this population are limited due to the rarity of LAM.

In our study, based on more than 800 LAM patients in our cohort, we aimed to analyze the risk of malignancy in LAM patients. That information would be of great help in determining whether we should incorporate the cancer screening plan for LAM patients.

## Methods

### Study population

This was a historical cohort study. We included patients who were registered in the LAM-China (NCT03193892) registry at Peking Union Medical College Hospital from May 2017 to July 2024. All patients who participated in the study signed an informed consent form. Patients who meet either of the following two conditions were included in the study: (1)patients who met the European Respiratory Society (ERS) diagnostic criteria for definite LAM or probable LAM. (2) patients who met the definite LAM diagnostic criteria of the American Thoracic Society (ATS)/Japanese Respiratory Society (JRS) [2, 10].

### Follow-up and data collection

Patients’ medical history, demographic data, CT images, 6-minute walking test (6MWT), pulmonary function test, and blood sample were collected when the patients were registered in the cohort. All the blood samples collected from patients are stored in the Clinical Biobank(ISO 20387), Peking Union Medical College Hospital, Chinese Academy of Medical Sciences.Patients were asked to return to the hospital for annual follow-up. Serum vascular endothelial growth factor-D (VEGF-D) levels were tested at every follow-up. As of July 2024, 556 patients had completed at least one follow-up visit. The same evaluations were conducted annually to evaluate the progression of the disease. The follow-up time for a patient was calculated from the LAM diagnosis time point to her last follow-up or the occurrence of outcome events, including death and a new diagnosis of malignancy.

The medical history of malignancy, such as the time of diagnosis, tumor site, treatment and pathological findings, was collected at each follow-up. For the incidence calculation, an event was defined as a diagnosis of malignancy occurring after or concurrently with the LAM diagnosis. “Concurrently” referred to malignancies diagnosed within 3 months prior to LAM diagnosis. Tumors in situ are not included.

### Statistical analysis

The standardized incidence ratio (SIR) was used to determine the relationship of cancer incidence between LAM patients and the general population. The cancer incidence data of the general population, as well as sex-specific and age-specific cancer incidence data, were obtained from the China National Central Cancer Registry (NCCR) [11]. Such nationwide incidence data was collected from 700 tumor registries covering all 31 provinces except Hong Kong, Macau and Taiwan, with a population of 52,3160,249 people, or 37.22% of China’s total population in 2018 [11]. It is also the most recent data available [11]. The person-year data are the sum of the follow-up times of all included LAM patients in our cohort. We assume that the occurrence of malignancy in LAM patients follows a Poisson distribution and conduct a two-tailed hypothesis test for the SIR with a null hypothesis of SIR = 1. A p value < 0.05 was considered significant, and the 95% confidence interval of the SIR was calculated via the exact method. All calculations were carried out via the *poisson.test()* function in R Studio (R version 4.4.0), which uses the Clopper‒Pearson method to calculate the exact confidence level [12].

## Results

### Status of malignant tumor occurrence in the LAM-China cohort

**Fig. 1:**
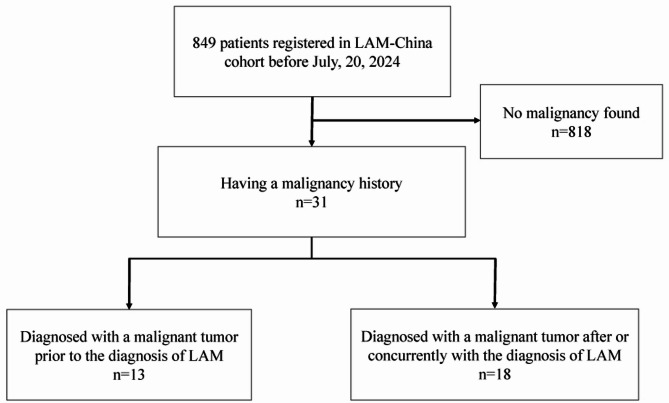
Flowchart of the study

**Fig. 2:**
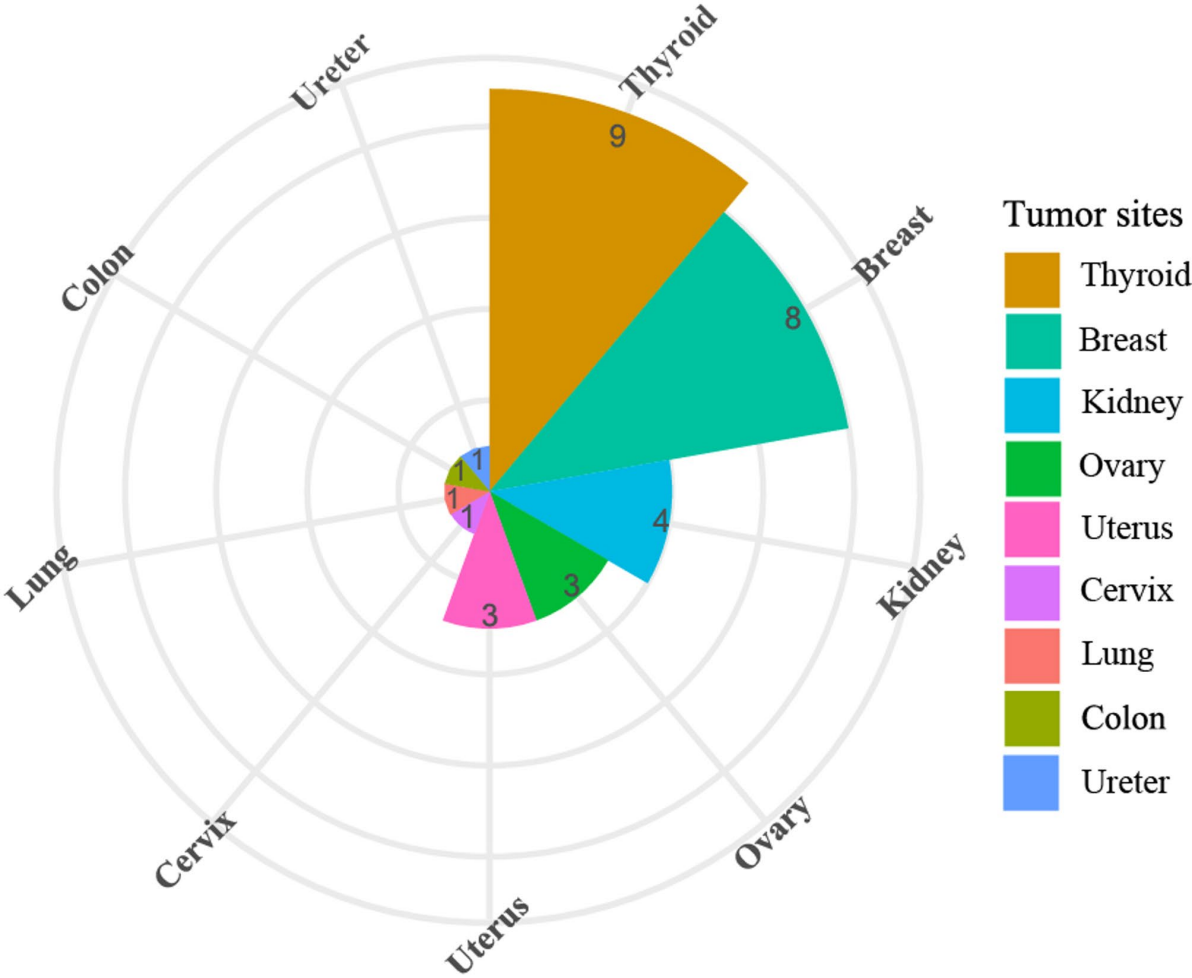
Types of malignant tumors and the number of cases that occurred in the LAM-China cohort

By July 20, 2024, a total of 849 people were included in this study, including 760 S-LAM and 89 TSC-LAM. The patients in the cohort are from 28 provinces of mainland China. 60% of them are from the eastern areas of China, including 127 patients from Beijing city. The proportions of patients from the central and western regions were 25% and 15%, respectively. Thirty-one patients (3.65%) had a history of malignancy (Fig. 1). The characteristics of these 31 patients are presented in Table 1. The tumor sites included the thyroid, breast, ovary, uterus, cervix, kidney, lung, ureter, and colon. Thyroid cancer and breast cancer were the most common, with 9 and 8 cases diagnosed, respectively (Fig. 2). No one had malignant tumors of more than one kind. Thirty (97%) patients underwent surgery to remove the tumor. Ten (32%) patients received radiotherapy or chemotherapy. To date, no patients in our cohort died from malignant tumors. More than 80% of LAM and malignant tumors develop successively within 5 years, with a median time interval of 1.5 years. Eighteen (58%) patients were diagnosed with malignancy after or concurrently with LAM diagnosis. Only these 18 patients were included in the incidence calculation.

**Table 1:** Characteristics of all LAM patients with a history of malignancy in the cohort

Factor	N=31
Sex, n (%)	
Female	31(100)
Geographical distribution	
Eastern areas of China	60%
Central areas of China	25%
Western areas of China	15%
Age of the first occurrence of LAM-associate symptoms, year, median (range)	40 (18–71)
Age of diagnosis of LAM, year, median (range)	45 (27–71)
Age of diagnosis of malignant tumor, year, median (range)	44 (22–65)
LAM diagnosis, *n* (%)	
Definite LAM	23 (71.2)
Probable LAM	8 (25.8)
LAM subtype, *n* (%)	
Sporadic LAM	28(90.0)
TSC-LAM	3(10.0)
VEGF-D (pg/mL), median(range)	909 (130–8516)
Renal angiomyolipoma, *n* (%)	
Yes	9 (29.0)
No	22 (71.0)
Retroperitoneal tumor, *n* (%)	
Yes	5 (16.1)
No	26 (83.9)

Abbreviations: LAM, lymphangioleiomyomatosis; VEGF-D, vascular endothelial growth factor D; TSC, tuberous sclerosis complex

The characteristics of these 18 patients are presented in Table 2. The median ages at diagnosis of LAM and malignancies were 40.5 years and 45.5 years, respectively. The median value of serum VEGF-D was 1049.5 pg/ml. Six (33.3%) patients had renal angiomyolipomas and 4 (22.2%) patients developed retroperitoneal tumors. Five patients received sirolimus treatment before malignancy, with a median treatment duration of 6.83 years. Fourteen patients underwent surgery, and 5 patients received postoperative radiotherapy or chemotherapy. Fifteen patients provided us with their morphological diagnosis data of their malignancies, and other malignancy information was obtained from the medical history document provided by the patients.

**Table 2:** Detailed characteristics of LAM patients who developed malignancy after or concurrently with LAM diagnosis abbreviations: LAM, Lymphangioleiomyomatosis; F, female; VEGF-D, vascular endothelial growth factor-D; AML, angiomyolipoma

Sex	LAM subtype	LAM diagnosis type	Age of diagnosis of LAM	Age of diagnosis of cancer	Tumor site	VEGF-D	Renal AML	Retroperitoneal tumor	Chylo- thorax		Using sirolimus before malignancy	Years of using sirolimus before malignancy
F	S-LAM	Definite LAM	53	53	Kidney	8516	No	No	No	No		-
F	S-LAM	Definite LAM	56	56	Ovary	323	Yes	No	No	No		-
F	S-LAM	Definite LAM	41	43	Breast	429	No	No	No	No		-
F	S-LAM	Definite LAM	27	27	Lung	6807	No	Yes	Yes	No		-
F	S-LAM	Probable LAM	47	47	Uterus	756	No	No	No	No		-
F	TSC-LAM	Definite LAM	33	34	Thyroid	299	Yes	No	No	No		-
F	S-LAM	Definite LAM	40	43	Thyroid	6890	No	Yes	No	Yes		2.8
F	S-LAM	Definite LAM	36	39	Uterus	2128	No	Yes	Yes	Yes		2.9
F	S-LAM	Definite LAM	42	42	Ovary	427	Yes	No	No	No		-
F	S-LAM	Definite LAM	38	52	Ureter	3475	No	Yes	No	Yes		9.3
F	TSC-LAM	Definite LAM	40	49	Breast	1199	Yes	No	No	Yes		6.8
F	S-LAM	Definite LAM	45	46	Thyroid	476	No	No	No	No		-
F	S-LAM	Probable LAM	53	57	Ovary	130	No	No	No	No		-
F	S-LAM	Definite LAM	60	60	Breast	909	No	No	No	No		-
F	S-LAM	Definite LAM	34	34	Kidney	1149	No	No	No	No		-
F	S-LAM	Definite LAM	45	45	Thyroid	3712	Yes	No	No	No		-
F	S-LAM	Definite LAM	39	39	Thyroid	753	Yes	No	No	No		
F	S-LAM	Definite LAM	40	49	Breast	1092	No	No	Yes	Yes		7.3

### The risk of malignancy is greater in LAM patients than in the general population

The three most prevalent post-LAM cancers among patients were thyroid cancer (*n* = 5), breast cancer (*n* = 4) and ovarian cancer (*n* = 3). The SIRs of malignant tumors at different sites were calculated (Table 3). Almost all kinds of cancers were significantly different: thyroid cancer (SIR = 10.87, 95% CI 3.53–25.37, *p* = 0.0001), breast cancer (SIR = 5.95, 95% CI 1.62–15.24, *p* = 0.005), and ovarian cancer (SIR = 24.54, 95% CI 5.07–71.86, *p* = 0.0003). This difference in incidence was also observed for malignancies of the kidneys, uterus and ureter. The only cancer for which we did not find an increased incidence in LAM patients was lung cancer (SIR = 1.7, 95% CI 0.04–9.24; *p* = 0.44). This result is somewhat counterintuitive since LAM lesions tend to occur in the lung, yet it does not seem to be a risk factor for lung cancer.

**Table 3:** Number and incidence of different kinds of malignancies in the LAM-China cohort

Tumor site	Incidence rate per 100000 person-years	Our cohort					
		Person-years	Observed cases (*n*)	Expected cases (*n*)	SIR	*P* value	95%CI
Thyroid cancer	20.95	2194	5	0.46	10.87	0.0001	3.53–25.37
Breast cancer	30.35	2215.6	4	0.67	5.95	0.005	1.62–15.24
Ovary cancer	5.54	2207	3	0.12	24.54	0.0003	5.07–71.86
Kidney cancer	1.86	2209.1	2	0.04	48.67	0.0008	5.91-176.21
Uterus cancer	7.08	2178	2	0.15	12.97	0.0107	1.57–46.91
Lung cancer	26.54	2212.9	1	0.59	1.7	0.439	0.04–9.49
Ureter cancer	0.28	2210.9	1	0.01	161.54	0.006	4.22-928.61

Abbreviations: LAM, lymphangioleiomyomatosis; SIR, standard incidence ratio; CI, confidence interval

The next part of this study focused on the overall incidence of malignancy in LAM patients compared with that in the Chinese general population after standardization by age. The expected number of patients with other malignancies in addition to LAM in our cohort was 5.63, while 18 patients developed cancer. The SIR for malignancy in our cohort was 3.20 (95% CI 1.89–5.05, *p* = 0.00003) (Table 4). These results indicate that LAM patients face a greater risk of developing malignancies than the general population does. A statistically significant difference in cancer incidence was observed across several age groups under 50 years. However, no significant difference was observed in the age groups over 50 years. The overall SIR of patients over 50 years of age was 1.65 (95% CI 0.53–3.84, *p* = 0.24). This phenomenon indicated that the cancer risk of LAM patients over 50 years of age did not seem to differ from that of the general population. The risk of malignancy, which might be caused by LAM, is likely to be present in younger patients.

**Table 4:** Malignancy incidence of LAM patients, standardized by the age-specific incidence rate in China

Age groups		Incidence rate per 100,000 person-years (China, female)		Our cohort					
				Pearson-years	Observed cases(*n*)	Expected cases(*n*)	SIR	95%CI	*P* value
15–19		11.51		11.25	0	0.001	-	-	-
20–24		21.40		30.75	0	0.007	-	-	-
25–29		51.17		133.00	1	0.068	14.69	0.37–81.94	0.066
30–34		88.22		224.08	2	0.198	10.12	1.22–36.49	0.017
35–39		124.85		376.58	2	0.470	4.25	0.52–15.37	0.081
40–44		191.87		389.00	3	0.746	4.02	0.83-11.75^a^	0.040
45–49		295.54		372.42	5	1.101	4.54	1.47–10.60	0.006
50–54		423.3		233.75	2	0.989	2.02	0.24–7.31	0.260
55–59		417.92		218.25	2	0.912	2.19	0.27–7.92	0.232
60–64		583.35		145.58	1	0.849	1.18	0.03–6.56	0.572
65–69		697.00		28.83	0	0.201	-	-	-
70–74		803.31		5.67	0	0.046	-	-	-
75–79		924.98		4.50	0	0.042	-	-	-
Total		273.02		2173.67	18	5.630	3.20	1.89–5.05	0.00003

Abbreviations: LAM, lymphangioleiomyomatosis; SIR, standard incidence ratio; CI, confidence interval;

^a^The 95% CI was calculated via the Clopper‒Pearson method, which is a conservative method that guarantees that the confidence level meets or exceeds a specified threshold, such as 95%, but may produce a wider interval. In this study, when 0.025 < *p* < 0.05, the lower limit of the 95% CI crosses 1 even when *p* < 0.05

## Discussion

This is a single-center retrospective study analyzing the incidence of malignancy in a Chinese LAM cohort. The study provided valuable insights into overall malignancy risk in the LAM population. We used the recently published 2018 Chinese malignant tumor incidence data as the standard population published by NCCR [11]. In standard population, people in the eastern areas account for 40%, people in the central areas and the western areas account for 25%, and 35% respectively. However, in the LAM cohort, due to the location of our center, we have a bigger proportion of eastern patients (60%) and smaller proportion of western patients (15%). Despite this, our patients came from nationwide, containing 28 mainland provinces. Therefore, although such nationwide cancer data was not perfect, it is still the most suitable one for our cohort to be compared with.

In this study, thirty-one patients (3.65%) had a history of malignancy. Notably, more than 80% of LAM cases and other malignancies occurred with a time interval of no more than five years. This finding is in accordance with the regular pattern of the second primary tumor, which is that the incidence of developing another malignant tumor increases in the first 5 years after the diagnosis of a malignancy and then decreases afterwards [13]. An explanation for this phenomenon is that therapy for the first malignancy induces another type of malignancy, such as radiotherapy and chemotherapy. In our cohort, of the 13 patients whose malignancies occurred before LAM, only three patients received radiotherapy or chemotherapy after malignancy, and one patient received IL-2 immunotherapy.

Sirolimus therapy for LAM could have the potential to induce the occurrence of malignant tumors because of its immune-suppressive effects. The use of immunosuppressants has been shown to increase the incidence of malignancy in organ transplant recipients [14, 15]. Among our patients with malignancies, only 5 received sirolimus when they were diagnosed with another malignancy, with a median duration of drug use of 6.8 years (Table 2). Theoretically, immunosuppressant therapy indeed has the chance to increase the risk of malignancy. However, there was no evidence suggesting that this occurred in our cohort. In fact, a meta-analysis revealed that sirolimus lowers the risk of developing malignancy and skin cancer in kidney transplant patients [16]. Another meta-analysis also reported that the use of sirolimus after nonrenal solid organ transplantation did not increase the risk of skin cancer [17]. Several studies on post-transplantation sirolimus therapy revealed that sirolimus did not increase the incidence of skin cancer but decreased it instead [18–21]. Sirolimus was also proved to be effective in preventing hepatocellular carcinoma in mouse models [22]. Considering the relatively low proportion of sirolimus use among patients with malignancies in our cohort, we cannot attribute the high malignancy risk in LAM patients to the immunosuppressive effects of sirolimus.

With respect to the incidence calculation, only 18 of 31 patients whose LAM occurred ahead were included. Even so, the incidence of malignancy was still 2 times higher than that in the general population (SIR = 3.20). The three most common cancers in our cohort were thyroid cancer, breast cancer and ovarian cancer. Thyroid cancer is one of the most common malignancies in the population [23]. It is the only nonreproductive-origin cancer whose incidence is much higher in women than in men [24, 25]. However, the incidence of thyroid cancer in LAM patients has not yet been reported. Our cohort included five new cases of thyroid cancer, four of which were papillary cancer and one of which was medullary cancer. The incidence of thyroid cancer was 10.88 times of that in the general population (SIR = 10.88). The SIRs of breast cancer in LAM patients have been reported to be 2.81 and 3.51 in the previous studies [7, 8]. The SIR was 5.95 in our cohort, which is consistent with previous studies showing that LAM patients have a greater risk of breast cancer. The four breast cancer patients all underwent surgery and accepted subsequent radiotherapy or chemotherapy. The incidence of ovarian cancer in LAM patients has not yet been evaluated until now, which also showed a greater risk in our LAM cohort. The pathological types of the three ovarian cancer cases in our cohort were high-grade serous adenocarcinoma, clear cell carcinoma and adult granulosa cell tumors.

For other malignancies, although we also found relatively higher risk in LAM patients, the number of new cases was so small that even one case would significantly increase the incidence of the cohort. Therefore, more evidence is needed to confirm the incidence change. The two kidney cancer patients are all clear-cell carcinoma. The two uterus carcinoma patients had endometrial carcinoma and high-grade endometrial stromal sarcoma. The patient with ureteral cancer was unable to undergo surgical treatment due to poor lung function so that pathological tissue cannot be obtained. She then received local radiotherapy after multidisciplinary consultation.

The only malignancy that was not different between LAM patients and the general population was lung cancer, which is not consistent with the findings of a recent Japanese study [9]. Even with annual high-resolution chest CT scanning, the incidence of lung cancer in our cohort was approximately 45 cases per 100,000 person-years, close to that of Chinese females (46 cases per 100,000 person-years) [11]. However, the incidence of lung cancer in the Japanese LAM cohort reached 301 cases per 100,000 person-years, which was much higher than that in the Chinese female population [9].

Interestingly, although the incidence difference could be observed in several 5-year age groups, no differences were observed in age groups over 50 years. This finding might suggest that the additional cancer risk associated with LAM disappeared in patients over 50 years of age, around the natural menopausal age (48.39 ± 3.63) of Chinese females [26]. A similar phenomenon was also observed in Nuñez’s study, in which LAM patients who were under 50 years of age had a greater risk of developing breast cancer [7]. LAM is a disease predominantly in premenopausal women with a potential mechanism of estrogen involved [27, 28]. We assume that this phenomenon of increased risk of malignancy in LAM before 50 years old might be related to increased estrogen activity [7]. Estrogen exposure or increased activity has been assumed as a risk factor of breast cancer [29–31]. Supplement of estrogen in postmenopausal women may increase the risk of cancer development [31, 32] Previous studies have also shown that postmenopausal estrogen therapy could stimulate endometrial proliferation and increase the risk of endometrial cancer [33, 34]. At the same time, the risk of serous and endometrioid ovary cancer increased [35, 36]. Although the mechanisms of increased malignancy in LAM may be complicated, the mechanisms of estrogen should be a point that requires further investigation.

In search for other potential contributors to the increased risk of malignancy in LAM, we investigated relevant demographic, geographic, educational and clinical characteristics such as lung function, CT grades, VEGF-D levels, renal AMLs, and retroperitoneal tumors etc., we did not find any risk factor associated with increased malignancy risk in LAM (data not shown).

There are several limitations in this work. Although the sample size included in our study is relatively large, it was a single-center retrospective study. Owing to the rarity of LAM, the findings from this study require further validation in a multiple center prospective study with larger sample sizes. Also, we need be cautious about the interpretation of the data comparing the prevalence of malignancy in a cohort with a general population. Even one case difference would increase or decrease the estimation a lot. Exact matching the reference population is difficult as the extremely low prevalence of LAM in general population [37]. Also, the medical resources in eastern areas of China are relatively more abundant. Such higher accessibility to medical resources makes it easier to detect asymptomatic early-stage cancers, which could lead to an increase in cancer incidence [38, 39]. The main point from our study, however, is that the overall risk of malignancy in LAM patients would increase. Another potential limitation is that the age-standardized incidence of each specific cancer was not published; thus, we could only calculate their overall incidence without age standardization.

In conclusion, our research suggests that the risk of developing a second malignant tumors was increased in LAM patients than that in the general population. Patients who were under 50 years of age or who were diagnosed with LAM for less than 5 years may face an increased risk. For each kind of malignant tumors that occurred in our cohort, LAM patients also had a relatively higher risk except lung cancer. These findings highlight the need for surveillance and early detection strategies for malignancies in LAM patients, given their increased vulnerability to cancer development.

## Electronic supplementary material

Below is the link to the electronic supplementary material.

Supplementary Material 1

## Data Availability

The datasets generated and analyzed for this study are not publicly available considering participant privacy but are available from the corresponding author upon reasonable request.

## Declarations

### Statement

The abstract of this article has been accepted for POSTER presentation at the 2025 ATS International Conference. Currently, the relevant DOI and conference paper links have not been assigned.

## Abbreviations

AML: Angiomyolipoma
ATS: American Thoracic Society
CI: Confidence interval
CT: Computed tomography
ERS: European Respiratory Society
JRS: Japanese Respiratory Society
LAM: Lymphangioleiomyomatosis
MAPK: Mitogen-activated protein kinase
mTOR: Mammalian target of rapamycin
NCCR: National Central Cancer Registry
PI3K/Akt: Phosphatidylinositol 3-kinase/protein kinase B
SIR: Standardized incidence ratio
S-LAM: Sporadic LAM
TSC: Tuberous sclerosis complex
VEGF-D: Vascular endothelial growth factor-D
6MWT: 6-minute walking test
