# Supplementary material for: Increased malignancy risk in patients with lymphangioleiomyomatosis: findings from a Chinese cohort
Source: Orphanet J Rare Dis. 2025 May 31;20:263. doi: 10.1186/s13023-025-03834-w (PMC12126872; doi:10.1186/s13023-025-03834-w)
Supplement: Supplementary file 1 — Supplementary Material 1 [file 13023_2025_3834_MOESM1_ESM.docx]

**Supplementary Table 1.** Baseline characters of LAM patients with or without malignancy

| Factors | LAM patients with Malignancy | LAM patients without Malignancy |  |
| --- | --- | --- | --- |
|  | N=26 | N=96 | *P* value |
| Age, y, mean (SD) | 47 (8.3) | 46.4 (7.7) | 0.929^a^ |
| Education background, *n* (%)  High  Low | 12 (52.2)  11 (47.8) | 36 (50)  36 (50) | 1.000^b^ |
| Annual household income, *n* (%)  High  Low | 16 (69.6)  7 (30.4) | 30 (44.1)  38 (55.9) | 0.062^b^ |
| Geographical distribution, *n* (%)  Eastern areas of China  Central areas of China  Western areas of China | 17 (65.4)  6 (23.1)  3 (11.5) | 61 (63.5)  25 (26.0)  10 (10.4) | 0.948^b^ |
| Menopause reached, *n* (%) | 11(42.3) | 42(43.8) | 0.895^b^ |
| LAM, subtype, *n* (%)  Sporadic LAM  TSC-LAM | 24 (92.3)  2 (7.7) | 86 (89.6)  10 (10.4) | 0.966^c^ |
| CT scan severity grade, *n* (%)  Ⅰ  Ⅱ  Ⅲ | 12 (46.2)  6 (23.1)  8 (30.8) | 42 (44.7)  16 (17.0)  36 (38.3) | 0.693^b^ |
| Renal angiomyolipoma, *n* (%)  Yes  No | 7 (26.9)  19 (73.1) | 35 (36.5)  61 (63.5) | 0.500^b^ |
| Retroperitoneal tumor, *n* (%)  Yes  No | 18(18.8)  91(94.8) | 4 (15.4)  22 (84.6) | 0.914^c^ |
| VEGF-D(pg/mL), median (IQR) | 776(426.7-3475.0) | 780(480.8-1716.2) | 0.783^d^ |
| FEV_1_ (L), median (IQR) | 2.22(1.72-2.56) | 2.3(2.00-2.56) | 0.822^d^ |
| FEV1 %predict (%), median (IQR) | 85(76.3-104) | 91.9(74.6-101.7) | 0.4074^d^ |

Abbreviations: TSC, tuberous sclerosis complex; VEGF-D, vascular endothelial growth factor D; IQR, interquartile range; FEV_1_, forced expiratory volume in one second.

Propensity score matching was used to match the 26 LAM patients who suffered malignancy with 96 LAM patients who did not. The matching factors are: age, TSC, menopause status, and rapamycin using. Baseline information was compared after matching.

^a^Student t test

^b^ χ^2^ Test

^c^Fisher exact test.

^d^Wilcoxon rank-sum test.

**Supplementary Table 2.** Univariate conditional logistic regression of the factors

| Factors | OR | 95%CI | *P* value |
| --- | --- | --- | --- |
|  |  |  |  |
| Renal angiomyolipoma | 0.60 | 0.222-1.62 | 0.31 |
| FEV_1_ | 0.79 | 0.311-2.01 | 0.62 |
| CT scan severity grade | 0.85 | 0.491-1.46 | 0.55 |
| Retroperitoneal tumor | 0.852 | 0.243-2.98 | 0.80 |
| Education background | 0.92 | 0.58-1.45 | 0.71 |
| FEV_1_ % predict | 0.99 | 0.96-1.01 | 0.24 |
| VEGF-D | 1.00 | 1.00-1.00 | 0.28 |
| Geographical distribution | 1 | 0.524-1.91 | 1.00 |
| Annual household income | 1.08 | 0.716-1.62 | 0.73 |

Abbreviations: OR, odds ratio; CI, confidence interval; VEGF-D, vascular endothelial growth factor D; FEV1, forced expiratory volume in one second.
